# Nonsurgical Periodontal Therapy for Diabetes Patients with Periodontal Disease

**DOI:** 10.4317/jced.63729

**Published:** 2026-02-26

**Authors:** María José Moya-Villaescusa, Arturo Sánchez-Pérez, Marta Arráez-Monllor, Paloma Portillo-Ortega, Bibiana Mateos-Moreno

**Affiliations:** 1 Department of Dermatology, Stomatology, Radiology and Physical Medicine, Periodontics Unit, Faculty of Medicine and Dentistry, Universidad de Murcia, Morales Meseguer University Hospital, Murcia 30008, Spain; 2 Department of Endocrinology, Virgen de la Arrixaca University Hospital, El Palmar (Murcia), Spain

## Abstract

**Background:**

Diabetes mellitus (DM) and periodontitis are chronic conditions with a well-established bidirectional relationship. This interaction not only worsens periodontitis severity but also complicates glycemic control. We aimed to determine whether nonsurgical periodontal treatment reduces glycosylated hemoglobin (HbA1c) levels at 3 and 6 months in type II diabetic patients with periodontal disease.

**Material and Methods:**

In this sequential case-control study conducted at the University Dental Clinic in Murcia, Spain, we enrolled thirty diabetic patients. Participants were allocated to either a test group (receiving comprehensive periodontal treatment with scaling and root planing) or a control group (receiving supragingival plaque removal only). Both groups received oral hygiene instructions. We evaluated periodontal parameters (HI, GBI, PPD, CAL) and HbA1c levels at baseline, 3, and 6 months, analyzing data with repeated-measures ANOVA and Bonferroni correction.

**Results:**

The periodontal treatment group demonstrated statistically significant reductions in HbA1c levels at both 3 and 6 months post-treatment (p &lt; 0.05). In contrast, we observed no significant changes in the control group.

**Conclusions:**

Our findings indicate that periodontal disease associates with elevated HbA1c levels in diabetic patients. Nonsurgical periodontal treatment significantly reduces both periodontal inflammation and HbA1c levels at 3 and 6 months, supporting its integration into comprehensive diabetes management.

## Introduction

Periodontal diseases (PDs) represent chronic inflammatory conditions triggered by bacterial biofilms, primarily manifesting as gingivitis and periodontitis (1). Multiple factors influence their development and progression, including tobacco use, genetic predisposition, and systemic conditions like diabetes mellitus (DM), all of which can compromise host immune response. Notably, advanced periodontitis (stages III and IV) can initiate or exacerbate various systemic conditions, particularly DM (2). A well-recognized bidirectional relationship exists between these conditions (3), mediated through advanced glycation end products (AGEs) and their receptors (RAGEs), which disrupt normal immune function (4 , 5). This interaction impairs endothelial and neutrophil activity, activates inflammatory cytokines, and hinders tissue repair processes (6). The systemic inflammation resulting from periodontitis may promote glucose intolerance and complicate glycemic control, as reflected in elevated glycosylated hemoglobin (HbA1c) levels (7 , 8). Nonsurgical periodontal treatment (NSPT) effectively controls periodontal disease (9), with some evidence suggesting it can improve glycemic control comparably to adding a second antidiabetic medication (10). Consequently, periodontal disease management through NSPT may play a vital role in overall diabetes care. This case-control study aimed to determine whether NSPT in type II diabetic patients with periodontal disease, compared to supragingival prophylaxis alone, influences HbA1c levels as the primary outcome. We also assessed hygiene index (HI), bleeding on probing (BOP), periodontal probing depth (PPD), and clinical attachment level (CAL) as secondary variables.

## Materials and Methods

1. Institutional Review Board Statement The Ethics Committee of Virgen de la Arrixaca Hospital, Murcia, Spain approved this study (ID: 141/2013). We conducted the research in accordance with the Declaration of Helsinki and registered it at ClinicalTrials.gov (NCT06506370). The study follows the STROBE guidelines for case-control studies. 2. Sample size calculation Based on the meta-analysis by Sgolastra et al. (10), we calculated that 15 patients per group would provide 80% power to detect an HbA1c difference of 0.25% at =0.05, accounting for a 30% dropout rate. This sample size aligns with previous comparable studies (11 , 12). 3. Patient recruitment and eligibility We recruited thirty type II diabetic patients referred from Virgen de la Arrixaca University Hospital to the University Dental Clinic, dividing them into periodontal (test, n=15) and non-periodontal (control, n=15) groups. Inclusion criteria: DM diagnosis; age 18 years; for the test group, moderate/advanced periodontitis (CAL 2 mm interproximally or 3 mm buccal/lingual in 2 non-adjacent teeth) and HbA1c between 5.5-11%. Exclusion criteria: Previous periodontal treatment; antibiotic use within the prior month; uncontrolled DM; pregnancy/lactation; unwillingness to provide informed consent. 4. Clinical parameters and calibration A single calibrated clinician (B.M-M.) performed all periodontal examinations (kappa=0.87). We recorded the following parameters at baseline, 3 and 6 months: Hygiene Index (HI) and Gingival Bleeding Index (GBI) on four surfaces Periodontal Probing Depth (PPD) and Clinical Attachment Level (CAL) on six surfaces per tooth Initial screening included orthopantomography. 5. Diabetes parameters and group assignment An endocrinologist assessed endocrine-metabolic variables (BMI, LDL, HDL, TG, HbA1c). Diabetes treatment regimens remained unchanged throughout the study. We defined periodontitis as CAL 2 mm interproximally or 3 mm buccal/lingual in 2 non-adjacent teeth. 6. Periodontal therapy Both groups received oral hygiene instructions and supragingival plaque removal. The test group additionally underwent nonsurgical periodontal treatment (NSPT) with subgingival scaling and root planing using Gracey curettes for pockets exceeding 4 mm. 7. Hypotheses and statistical analysis Null hypothesis: NSPT does not affect HbA1c control in diabetic patients with periodontitis. Alternative hypothesis: NSPT improves glycemic control (reduces HbA1c) at 3 and 6 months. The primary variable (HbA1c) demonstrated normal distribution (Shapiro-Wilk test). We employed two-factor ANOVA with repeated measures for analysis.

## Results

1. Sample characteristics and homogeneity The study groups demonstrated homogeneity at baseline for all recorded variables, including demographic characteristics, diabetes parameters, BMI, and biochemical markers (LDL, HDL, TG, HbA1c). We found no statistically significant differences between groups for any variable at baseline, 3 or 6 months (Table 1).

Table 1

2. Evolution of periodontal and metabolic parameters We observed a significant time-by-group interaction for both primary and secondary outcomes (Table 2).

Table 2

**Figure 1:**
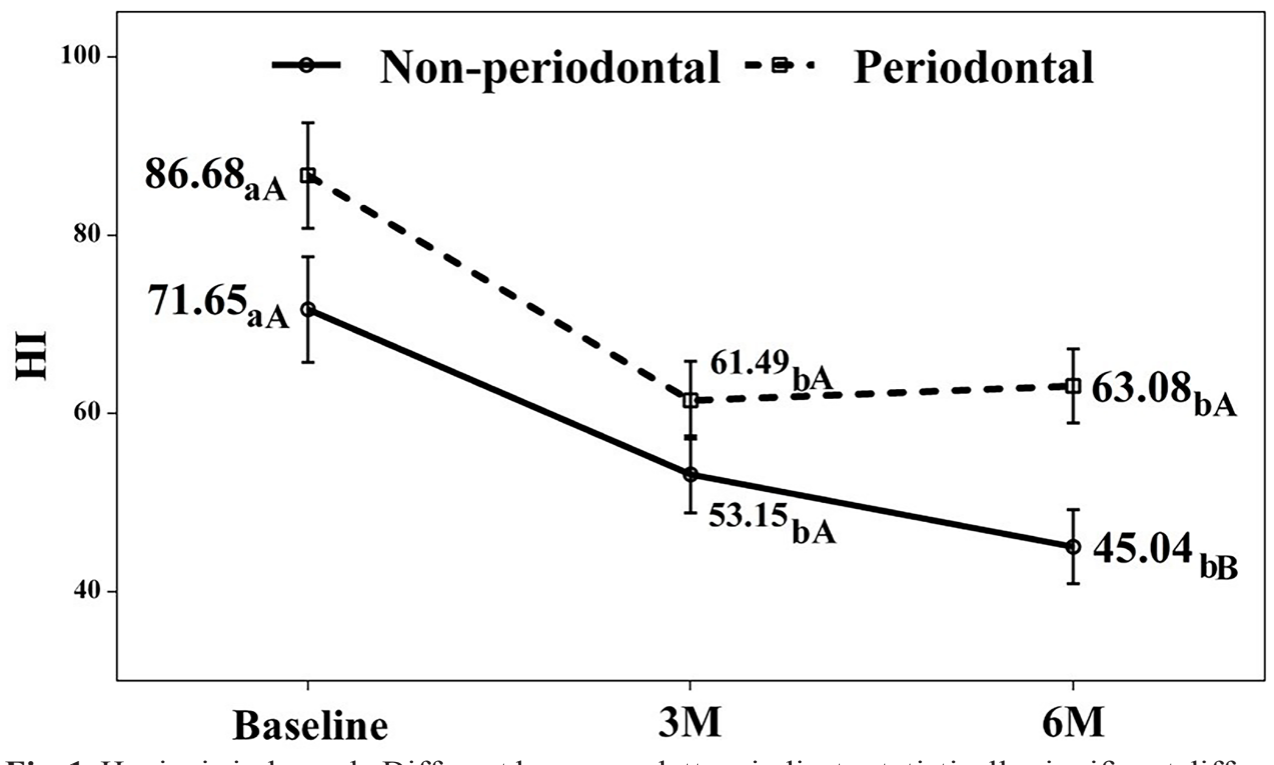
Hygienic index. a-b. Different lowercase letters indicate statistically significant differences between time points in the same group (Bonferroni correction). A-B. Different capital letters indicate statistically significant differences between groups at the same time point (Bonferroni correction).

**Figure 2:**
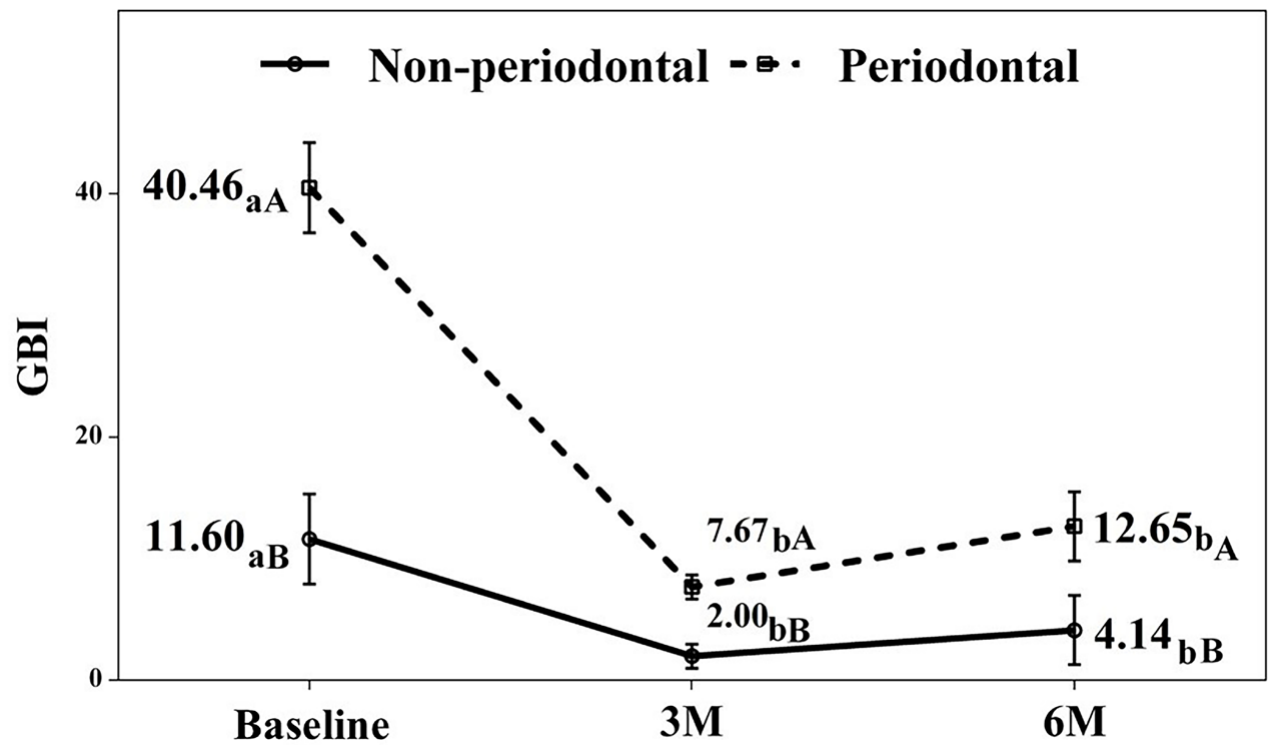
Gingival bleeding index. a-b. Different lowercase letters indicate statistically significant differences between time points in the same group (Bonferroni correction). A-B. Different capital letters indicate statistically significant differences between groups at the same time point (Bonferroni correction).

The detailed progression for each variable follows. Hygiene Index (HI) and Gingival Bleeding Index (GBI) Both groups showed significant improvement in HI and GBI at 3 and 6 months compared to baseline (p &lt; 0.05; Figs. 1,2).

1

2

**Figure 3:**
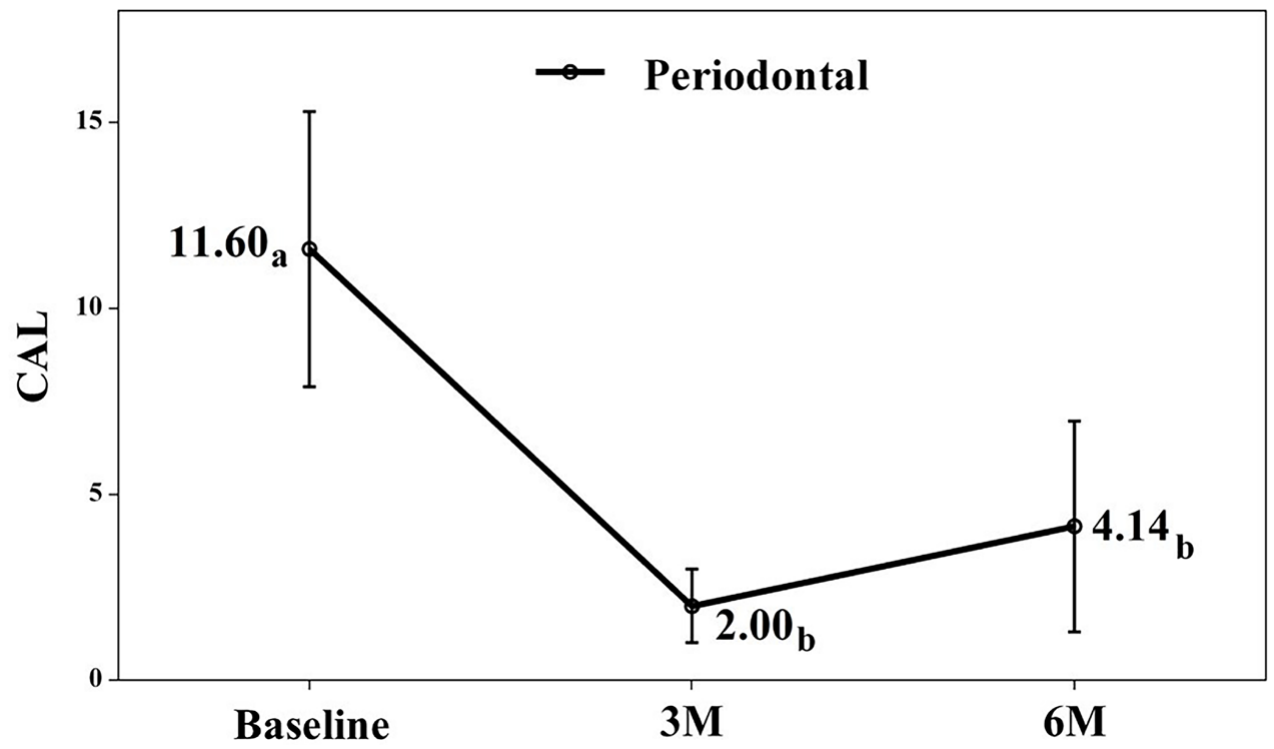
Clinical attachment level. a-b. Different lowercase letters indicate statistically significant differences between time points in the same group (Bonferroni correction). A-B. Different capital letters indicate statistically significant differences between groups at the same time point (Bonferroni correction).

The test group, which began with poorer indices, demonstrated particularly substantial improvement. While we found no significant differences between 3- and 6-month values for HI in either group, the test group's GBI showed a slight but significant increase between these time points. Clinical Attachment Level (CAL) As anticipated, the control group exhibited no clinical attachment loss. The test group, however, showed significant attachment gain (&gt;1 mm) at 3 months (mean CAL: 3.16 mm, p = 0.001) that persisted at 6 months (mean CAL: 3.24 mm, p = 0.001), with no significant difference between the two follow-up periods (Fig. 3).

3

**Figure 4:**
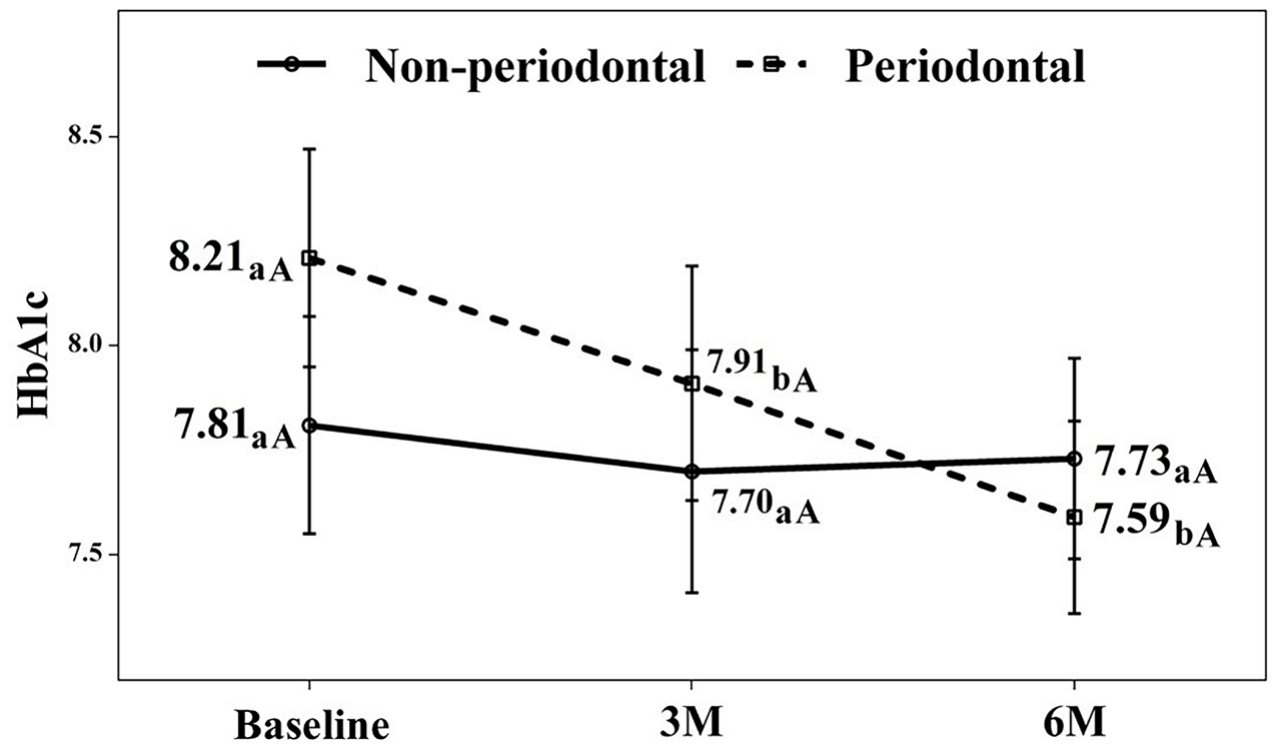
Glycosylated hemoglobin (HbA1c) levels. a-b. Different lowercase letters indicate statistically significant differences between time points in the same group (Bonferroni correction). A-B. Different capital letters indicate statistically significant differences between groups at the same time point (Bonferroni correction).

Glycosylated Hemoglobin (HbA1c) The control group maintained stable HbA1c levels throughout the study. Conversely, the test group exhibited statistically significant HbA1c reductions at both 3 months (mean reduction: 0.3%, p = 0.047) and 6 months (mean reduction: 0.62%, p = 0.005) compared to baseline.The difference between 3- and 6-month reductions was not statistically significant (Fig. 4).

4

## Discussion

Our study demonstrates that nonsurgical periodontal treatment (NSPT) significantly improves both periodontal health and glycemic control in type II diabetes patients. The 0.62% reduction in HbA1c at 6 months in the treatment group, combined with the absence of improvement in controls, highlights the clinical importance of periodontal therapy in diabetes management. 1. Primary outcome: Glycemic control The significant HbA1c reduction following NSPT agrees with several systematic reviews and meta-analyses (13 , 14), which report comparable reductions of 0.27-0.56%. Our results align particularly well with a recent review (15), confirming NSPT efficacy at both 3 and 6 months. The control group's lack of HbA1c improvement despite receiving supragingival prophylaxis and oral hygiene instruction suggests that subgingival periodontitis components primarily drive the systemic inflammation affecting glycemic metabolism. This interpretation finds support in Chen et al. (16), who reported that patients with milder periodontal damage gain minimal metabolic benefit from superficial debridement. Although some large trials, such as that by Engebretson et al. (17), found no significant effect, this discrepancy may stem from variations in baseline HbA1c, periodontal disease severity, and post-therapy supportive care intensity. Our consistent improvements at both 3 and 6 months emphasize the value of meticulous supportive periodontal therapy in sustaining metabolic benefits. 2. Secondary outcomes and mechanisms The substantial improvements across all clinical periodontal parameters confirm NSPT's local effectiveness in diabetic patients, consistent with previous reports (9 , 18). The bidirectional periodontitis-diabetes relationship involves chronic inflammation and advanced glycation end products (AGEs) (3 - 5). Periodontitis contributes to a systemic proinflammatory state (19), that can increase insulin resistance, while hyperglycemia and AGE accumulation may exacerbate periodontal tissue destruction (3 , 4), creating a self-perpetuating cycle. Our finding that patients with higher baseline HbA1c had more severe periodontitis supports this pathophysiological model. 3. Limitations and strengths Study limitations include the sample size and single-center design, which may affect generalizability. Using orthopantomography rather than full-mouth radiographs, while practical for screening, might have limited early bone defect detection. The non-randomized design potentially introduces selection bias. However, strengths include the examiner's rigorous calibration and the homogeneous, well-characterized sample. The study design also reflects real-world clinical practice where withholding treatment from periodontitis patients raises ethical concerns. 4. Clinical implications and conclusion Notwithstanding these limitations, this pragmatic study provides locally relevant evidence to support the establishment of a standardized periodontal treatment protocol for this specific patient population within our university clinic setting, with the ultimate goal of facilitating structured interdisciplinary co-management with Endocrinology. The collaboration between endocrinology and periodontology units proved fundamental to this study. Our results support integrating periodontal examination and NSPT into standard care protocols for type II diabetes patients. Periodontitis management represents not just an oral health concern but a valid approach to improving glycemic control and potentially reducing diabetic complications.

## Conclusions

This study provides evidence that nonsurgical periodontal treatment significantly improves both periodontal health and glycemic control in type II diabetes patients, as shown by HbA1c reductions at 3 and 6 months. Integrating periodontal examination and treatment into standard diabetes management should be considered a valuable strategy for enhancing metabolic outcomes and overall patient health.

## Figures and Tables

**Table 1:** Distribution and characteristics of the sample (all patients were diagnosed with type II DM.)

Characteristics	Test group: DM + Periodontal		Control group: DM + No periodontal	
	Mean ± SD	Min–Max	Mean ± SD	Min–Max
Age (years)	55.59 ± 10.64	33.37–69.4	54.97±8.60	40.00–66.30
Duration of Disease (years)	18.13 ± 10.10	4.00–39.00	18.87±12.70	4.00–42.00
BMI	29.24 ± 4.32	20.52–41.9	30.52 ±3.74	25.82–38.10
HDL	61.23 ± 18.99	25.00–93.00	50.13 ± 10.24	33.00–71.00
LDL	91.08 ± 16.49	54.00–124.00	100.47±29.64	44.00–159.00
TG	94.62 ± 54.71	33.00–238.00	126.73±58.00	51.00–218.00
HbA1c	8.21 ± 1.22	5.90–10.80	7.81±0.61	6.80–9.20
Sex	Male 11	Female 4	Male 11	Female 4

Abbreviations: BMI: body mass index; HDL: high-density lipoprotein; LDL: low-density lipoprotein; TG: triglyceride; HbA1c: glycosylated hemoglobin; Max: maximum value; Min: minimum value; SD: standard deviation.

**Table 2:** Evolution of the periodontal variables over time and the time per subject.

Variables	Time			Within-subject effects			
	Baseline	3 M	6 M	Time		Group* Time	
	Mean ± SD	Mean ± SD	Mean ± SD	F (2;56)	p (Î·2)	F (2;56)	p (Î·2)
GBI				49.501	< 0.001 (0.639)	15.366	< 0.001 (0.354)
Nonperiodontal	11.60 ± 10.54	2.00 ± 2.11	4.14 ± 5.09				
Periodontal	40.46 ± 17.32	7.67 ± 5.00	12.65 ± 14.70				
HI				22.842	< 0.001 (0.449)	0.756	0.474 (0.026)
Nonperiodontal	71.65 ± 25.40	53.15 ± 15.56	45.04 ± 14.69				
Periodontal	86.68 ± 20.22	61.49 ± 17.88	63.08 ± 17.44				
HbA1c				4.466	0.016 (0.142)	2.803	0.069 (0.094)
Nonperiodontal	7.81 ± 0.64	7.70 ± 0.87	7.73 ± 0.65				
Periodontal	8.21 ± 1.23	7.91 ± 1.27	7.59 ± 1.09				

Abbreviations: GBI: gingival bleeding index; HI: hygienic index; HbA1c: glycosylated hemoglobin.Notes: We summarized the values of the means, their standard deviations, the values of the eta squared, and the time factors per subject.

## Data Availability

The data of present study are available to readers in Excel upon request to the corresponding author (arturosa@um.es).
